# Non-papillary serous cystadenoma of the epididymis associated with infected hydrocele of the testis: A case report

**DOI:** 10.1016/j.heliyon.2024.e29612

**Published:** 2024-04-16

**Authors:** Takashi Ando, Yoshiharu Fukuhara, Naoto Miyanaga, Haruo Ohtani

**Affiliations:** a Departments of Urology, Mito Saiseikai General Hospital, Ibaraki, Japan; b Departments of Pathology, Mito Saiseikai General Hospital, Ibaraki, Japan

**Keywords:** Serous cystadenoma of the epididymis, Case report

## Abstract

A 50-year-old Japanese man with enlargement of the right scrotum was presented to our hospital. Preoperative examination confirmed a multilocular cyst with septa attached to the testis. Radical orchiectomy was performed. Pathological examination revealed closely-located two cysts; larger one was infected hydrocele testis, and smaller one was epithelial cyst, which were immunohistochemically positive widely for estrogen receptor (ER) and partly for progesterone receptor (PR). We concluded that the smaller cyst was serous cystadenoma of the epididymis.

## 1 Introduction

Cystadenoma of the epididymis is a benign epithelial tumor that arise from epididymal ducts which is usually located in the caput epididymis [1]. Pure (non-papillary) serous cystadenoma is rarely seen. To the best of our knowledge, only 5 reports documented 6 cases of serous cystadenoma in the epididymis [[2], [3], [4], [5], [6]]. This case report describes the clinical presentation and gross and microscopic features of this tumor.

## 2 Case presentation

**Fig. 1:**
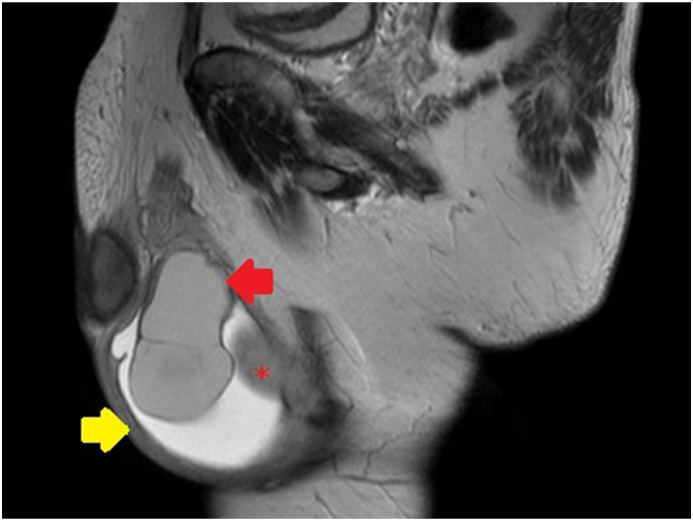
Pre-operative magnetic resonance Images (MRI) Sagittal images of MRI. Testis and testicular adnexa were in the space filled with fluid (Yellow arrow). This room was the 7cm size cyst in the resected specimen after orchiectomy. The red star was the right testis. Multinodal cysts without solid lesion were attached to the epididymis (Red arrow). The wall of the cysts were not contrast-enhanced. The inner content of the cysts was consistent with hematoma.

**Fig. 2:**
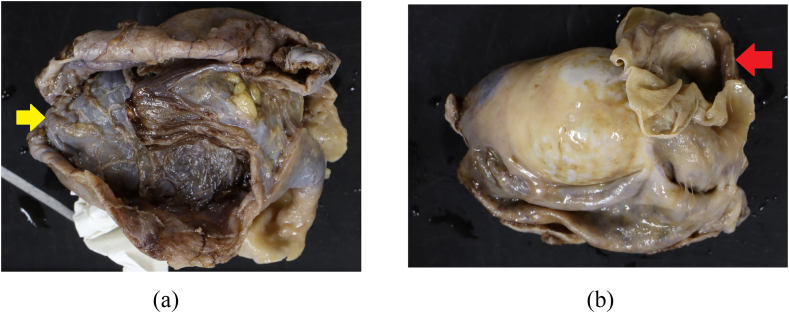
Gross examination of the resected right epididymis and testis 2A – The bigger cyst was 7.0 cm in size (Yellow arrow). The thick wall of the cyst indicated that the wall had inflammation. Pathological diagnosis was hydrocele with inflammation. 2B – The smaller cyst was attached in the epididymis, which appeared after opening the bigger cyst. The size was 3.0 cm (Red arrow).

**Fig. 3:**
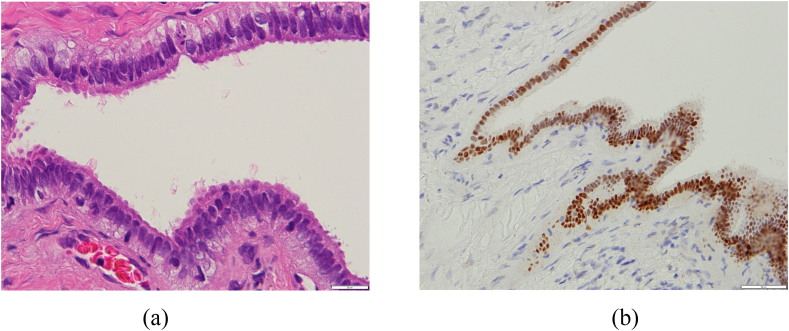
**Pathological findings of the serous cystadenoma of the epididymis**. 3A– The cystic lining epithelium consists of a single layer of cuboidal to columnar serous cells. Some are ciliated. (Hematoxylin and eosin, original magnification × 400) 3B– The cystic lining epithelium is strongly positive for estrogen receptors. (Original magnification × 400).

A 50-year-old Japanese man, non-smoker was referred to our hospital presenting swollen right scrotum. It was rapidly growing in size without any local pain. It had been normal size until 3 months before visiting the prior hospital. He had undergone the puncture there. The cyst content was red color fluid and he was referred to our hospital. The patient denied trauma to the testicular/perineal bump or history of infections including sexually transmitted diseases. Medical history was hypertension for which the patient took an angiotensin receptor blocker (ARB) tablet once daily. He had no known allergies. Ultrasonography of the scrota revealed a multilocular cyst with septa attached to the testis without abnormalities in the testis. Preoperative Magnetic Resonance Image (MRI) scan revealed that the scrotum had fluid-filled sac (which was described as the bigger cyst, 7 cm in size, in the pathological examination) and multinodal cyst without solid lesion (Fig. 1). There was no contrast enhanced rim on the cyst. The contents of the cyst had high-intensity on T1-weighted image and relatively low-intensity on T1-weighted image which was consistent with hematoma. The patient chose to be treated by radical orchiectomy. The gross pathological examination demonstrated two cysts, 7.0 cm and 3.0 cm in size, attached to the right testis (Fig. 2). The larger one was diagnosed as hydrocele with inflammation on the wall. The smaller one was derived from the right epididymis which was closely located cranially to the larger cyst. Microscopic examination revealed that the smaller cyst was covered by single row of bland tall columnar cells, which were immunohistochemically EpCAM(+), BerEP4(+), calretinin(−), D2-40(−), WT-1(+), estrogen receptor (ER)(+)(nearly 100 %) and progesterone receptor (PR)(+)(approximately 20 %) (Fig. 3). No intracellular mucin, papillary growth or stromal invasion were found. With these findings the smaller cyst was diagnosed as serous cystadenoma of the epididymis, which categorized in the ovarian type tumors of the collecting ducts and rete testis as described in the WHO classification [1].

The patient was discharged without any complications. He visited our hospital several times to assess the post-operative condition after the discharge. No recurrence or complication had been reported.

## 3 Discussion

Serous cystadenoma of the epididymis is described in the chapter 7 "Tumors of the testicular adnexa" in 2022 WHO classification. It is categorized as one type of ovarian-type cystic tumors [1]. Ovarian-type cystic tumors of the collecting ducts and rete testis are classified into serous cystadenoma, serous tumor of borderline malignancy, serous cystadenocarcinoma, mucinous cystadenoma, mucinous borderline tumor, mucinous cystadenocarcinoma, as it is in the case of cystic tumors in the ovary. Other than these tumors, cystadenoma of the epididymis and papillary cystadenoma of the epididymis can be found as cystic lesions in WHO classification. Since the first report of papillary cystadenoma of the epididymis in 1956, all case reports were cystic lesions with papillary formations until pure (non-papillary) serous cystadenoma was first reported by Pich and Galliano in 2005 [3]^(4)^. “Papillary” cystadenoma of the epididymis has been drawn attention to could-be manifestation of von Hippel-Lindau syndrome (VHL). Lesions are usually arising from the head of the epididymis and can present as a cystic lesion with a thick wall and several peripheral solid nodules with increased vascularity. Although most of these cystic tumors are benign, there have been reports of borderline. Young and Scully reviewed 14 cases of testicular and paratesticular tumors of ovarian common epithelial type, all of them were either serous papillary or mucinous cystadenomas of borderline malignancy [7]. Since the first report of non-papillary serous cystadenoma of the epididymis was published [2], 4 reports about 5 cases have been published, the report about 83 year old male [3], case of 64 year old male [4], report of 2 cases, 12 year old and 39 year old [5] and the case of bilateral serous cystadenoma [6]. The serous cystadenoma is ovarian-type epithelial tumors of the testis, which is believed to originate from remnants of Müllerian ducts in epididymis or from Müllerian metaplasia of the mesothelium of the tunica vaginalis of the testis. Clinical features are symptoms related to mass effects. Chief complaint of the cases in previous reports was scrotal swelling or testicular mass [[2], [3], [4], [5], [6]]. In our case the inner contents of the bigger cyst (hydrocele testis) was bloody fluids. We guessed that clinical manifestation of our case of rapid growing in size in the right scrotum was the result of incidental infectious bleeding in the hydrocele testis, and was not related to the serous cystadenoma. Both the gross and microscopic features were similar to those of ovarian serous cystadenoma [2]. The tumors are cystic and contain serous fluids. The cysts were lined by a non-stratified cuboidal to ciliated columnar epithelium without atypia or mitotic figures [1]. Tumor resection is curative. This benign neoplasm can be either in a unilateral or bilateral fashion, wherein bilateral lesions imply genetic association. The differential diagnosis of paratestiicular tumors includes cystadenomas/carcinomas of the rete testis, mesothelioma, adenomatoid tumors, papillary cystadenomas and metastatic lesions. Two major differential diagnosis of serous cystadenoma is considered to be spermatocele and adenomatoid tumor, both of which are negative of ER and PR. Spermatocele can be discarded by checking the lack of spermatozoa. Spermatocele is positive for CD10 and negative for cytokeratin and S-100 [5]. Adenomatoid tumor is usually solid rather than cystic, cilia are rarely seen [2]^(5)^. It is a small, firm, rather solid and circumscribed lesion with epithelial-like cells with prominent vacuoles [2].

## 4 Conclusion

We report a case with serous cystadenoma of the epididymis, which should be considered as differential diagnosis of paratesticular cyst tumor.

## Consent

Written informed consent was obtained from the patient.

## FUNDING

This research did not receive any specific grant from funding agencies in the public, commercial, or not-for-profit sectors.

## CRediT authorship contribution statement

**Takashi Ando:** Conceptualization. **Yoshiharu Fukuhara:** Supervision. **Naoto Miyanaga:** Supervision. **Haruo Ohtani:** Supervision.

## Declaration of competing interest

The authors declare that they have no known competing financial interests or personal relationships that could have appeared to influence the work reported in this paper.
